# Clinical exercise therapy program with multiple myeloma patients: Impacts on feasibility, adherence and efficacy

**DOI:** 10.1007/s00520-022-07369-9

**Published:** 2022-10-03

**Authors:** Michael Mendes Wefelnberg, Timo Niels, Udo Holtick, Franziska Jundt, Christoph Scheid, Freerk T. Baumann

**Affiliations:** 1 Department I of Internal Medicine, Center for Integrated Oncology Aachen Bonn Cologne Dusseldorf, University Hospital Cologne, Kerpener Str. 62, 50937 Cologne, Germany; 2 Medical Clinic and Polyclinic II, Center for Internal Medicine, University Hospital Wuerzburg, Oberdürrbacher Straße 6, House A3, 97080 Würzburg, Germany

**Keywords:** Multiple myeloma (MM), Feasibility, Adherence, Efficacy

## Abstract

**Purpose:**

Multiple myeloma (MM) is a severe hemato-oncological disease with high mortality and increasing incidence rate. Since evidence on exercise therapy in MM patients remains limited, this study examines feasibility, adherence, and efficacy based on real-life data from an oncologic care structure.

**Methods:**

A data evaluation of MM patients who participated in the oncologic exercise and movement therapy (OTT) at the Cologne University Hospital between 2012 and 2019 was conducted. The patient flow was incrementally reduced to four cohorts, intention-to-treat cohort (ITTC), safety cohort (SC), adherence cohort (AC), and efficacy cohort (EC). Cohorts were evaluated descriptively and by means of correlation analysis as well as group and time comparisons.

**Results:**

Thirty patients registered at the OTT between 2012 and 2019 (ITTC). The SC (*N* = 26) attended exercise therapy on average about one session per week over a period of 8 months. One-third dropped out within 3 months. In the AC (*N* = 15), BMI at baseline exhibited a strong and very significant negative correlation with exercise adherence. In the EC (*N* = 8), a significant improvement in physical functioning and a tendency towards significance in fatigue reduction between two measurement points was observed. No adverse events were documented.

**Conclusions:**

The present observatory study reveals safety and feasibility while indicating adherence and efficacy of exercising MM patients under real-life therapy circumstances. Found obstacles to exercising as well as improvements in questionnaire scale scores need to be further examined in confirmatory study designs.

## Background

Multiple myeloma (MM) is the second most frequent hematological cancer entity with an increasing annual incidence and high mortality rate [1–3]. The most common symptoms of MM include disseminated osteolysis and, consequently, bone fragility, anemia, and hypercalcemia as well as renal dysfunction or even terminal renal failure [4]. Moreover, intensive drug regimens and radiation increase severe burden of fatigue and polyneuropathy and a significant reduction in physical capacity and health-related quality of life [2, 5, 6].

The feasibility, efficacy, and dosage of exercise therapy interventions has been extensively reported in oncologic conditions but remains limited in MM patients [7–11]. Exercise therapy interventions reduce side effects of medical treatment, enhance physical capacity, and reduce symptoms of anxiety and depression in patients.

Low quality intervention studies and qualitative studies indicate associations between exercise and positive outcomes in MM patients, such as improvements in physical capacity, strength, and quality of life [9, 12–15]. These previous studies systematically excluded patients with limitations for participation in exercise therapy, such as histories of or increased risks for bone lesions. Furthermore, only few reliable data exist on exercise adherence and attrition rates in MM patients, especially those reflecting real-life circumstances. Based on routinely collected data of an oncologic care structure, the following study examines the feasibility, adherence, and efficacy of exercise in MM patients under real-life therapy conditions.

## Methods

### Study design

The evaluation was conducted of the data of all patients with MM who participated in the in- and outpatient Oncological Exercise and Movement Therapy (OTT) at the Cologne University Hospital from January 2012 to October 2019. In order to reflect real-life circumstances, besides a minimum of one executed exercise session, no further inclusion or exclusion criteria were applied. The OTT provides an evidence-based and individually tailored exercise therapy for cancer patients before and during medical treatment. The training is performed on endurance machines and exercise machines for the major muscle groups complemented by symptom and side effect specific exercise modules. As the OTT is a registered and independently funded research project with approval by the Institutional Review Board of the Medical Faculty of the University of Cologne (N. 13–050), upon admission, patients consent to the use of their data for research purposes.

The data sources comprise of the digital patient database of the OTT, digitally and analogue archived data of the OTT, and the patient database of the Cologne University Hospital. The data include routinely collected attendance, demographic, medical, and cancer-specific questionnaire data along with data from strength and endurance tests. The attendance is registered by an electronic chip card system. As the first session usually consists of anamnesis, registration paperwork as well as introduction into the electronic chip card system and exercise machines, for evaluation purposes, it is not regarded as a completed exercise session. Questionnaire and physical assessments are usually conducted in predefined exercise session intervals of 12 weeks after a brief familiarization period of about 1 to 2 weeks. Endurance capacity is determined by a quasi-ramp protocol on a bicycle ergometer or cross-walker with an input load of 30 watts and an increase of 15 watts per increment, with a 1-min increment duration. Muscle strength is determined by eight repetitions maximum (8RM) tests at various stationary strength exercise machines. These include back extension, leg curl, abdominal crunch, rowing, bench press, and leg extension. The questionnaire assessment usually comprises the Cancer Quality of Life Questionnaire of the European Organisation for Research and Treatment of Cancer (EORTC QLQ_C30), the Multidimensional Fatigue Inventory (MFI), Global Physical Activity Questionnaire (GPAQ), and the Hospital Anxiety and Depression Scale (HADS). The EORTC QLQ_C30 consists of several single and multi-item scales that range in scores from 0 to 100. A high scale score represents a higher response level.

### Data preparation and statistical evaluation

Firstly, the patient flow was incrementally reduced into four cohorts to enable the assessment of feasibility, safety, exercise adherence, and efficacy, respectively. The intention-to-treat cohort (ITTC) contains all patients that have been registered at the OTT. The ITTC was descriptively analyzed regarding time of and reasons for dropout. The safety cohort (SC) consists of all patients that exhibit a minimum of two attendances or one completed exercise session respectively and was analyzed descriptively with respect to safety (adverse events), exercise adherence, and early dropout rate. Due to ad hoc determination, and in contrast to the common definition of early dropout from prescribed exercise regime [11], early dropout was calculated based on the effect and entity specific exercise recommendations for oncologic patients (i.e., a minimum of 12 weeks at a frequency of two to three sessions) [7]. If not otherwise documented, patients whose last session was less than 4 weeks ago were considered still attending. The adherence cohort (AC) includes all patients that exhibited complete medical, demographic, and questionnaire data sets, and facultatively physical assessment data, for the baseline assessments. Based upon these categories and the data extractable known baseline values associated with higher or lower exercise adherence were analyzed. Hereto, correlation analysis and group comparisons, preceded by normal distribution testing (Shapiro–Wilk), were conducted. All patients that participated in at least one consecutive assessment and exhibited complete medical, demographic, and questionnaire data sets, and facultatively physical assessment data, formed the efficacy cohort (EC) and were submitted to longitudinal statistical calculations, parametric or non-parametric, depending on the results of preceded normal distribution testing (Shapiro–Wilk). For all statistical calculations, IBM’s Statistical Package for the Social Sciences (SPSS), version 26, was utilized.

## Results

### Quantitative results

**Fig. 1:**
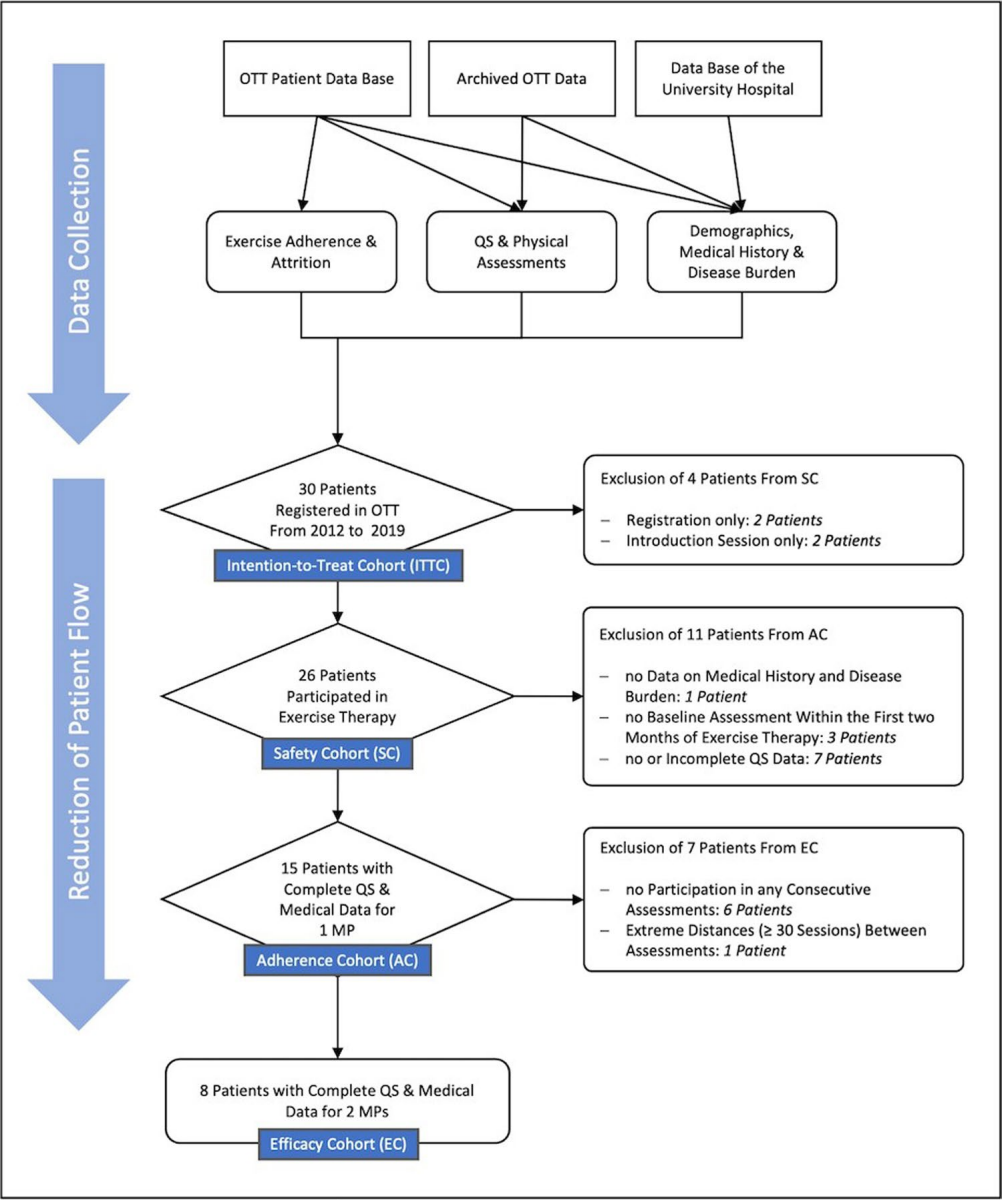
Quantitative results of data collection and resulting cohorts of patient flow reduction. Abbreviations: AC, adherence cohort; EC, efficacy cohort; SC, safety cohort; MP, measurement point; OTT, oncologic exercise and movement therapy; QS, questionnaire

**Fig. 2:**
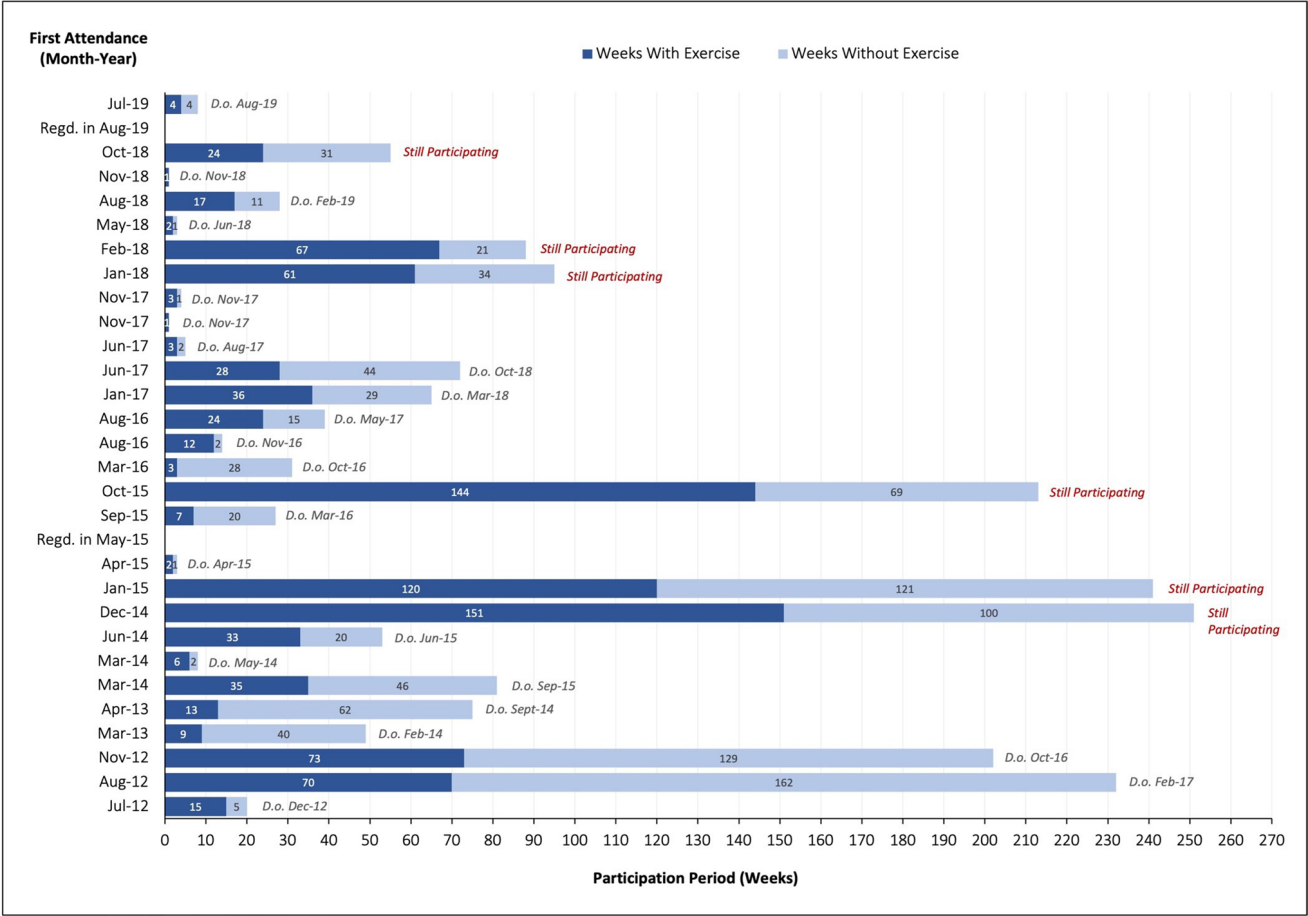
Patient flow and exercise participation of MM patients at the OTT between July 2012 and September 2019 (ITTC). Abbreviations: D.o., dropout; ITTC, intention-to-treat cohort; Regd., registered

Between July 2012 and September 2019, a total of 30 patients with MM were registered at the OTT. The application of the predefined cohort criteria yielded cohort sizes of *N* = 30 (ITTC), *N* = 26 (SC), *N* = 15 (AC), and *N* = 8 (EC). Due to dropout and non- or irregular participation in baseline and consecutive assessments, patients had to be excluded from statistical evaluation. For a total of nine patients, data on strength and endurance tests were available while six of whom inconsistently and varying between measurement points (MPs) performed a few or a single test only. Therefore, physical assessment data could not serve as cohort allocation and evaluation criterion. Merely, three patients provided evaluable data sets for three MPs, hence, calculations for three MPs were omitted. The complete patient flow and resulting cohorts are illustrated in Figs. 1 and 2.

Of the 30 patients in the ITTC, four patients have either never attended the OTT or only the first anamnesis and introduction session. As these patients did not commence exercise therapy and did not participate in any baseline assessment, they were excluded from any further evaluation.

The SC consists of 26 patients with a mean age of 64 years (SD =  ± 4) and a mean BMI of 26 (SD =  ± 5). On average, the patients suffer from five (SD =  ± 1.6) characteristic symptoms of multiple myeloma and/or medical treatment, which are, in descending order, disseminated bone lesions (69.2%), cancer-induced polyneuropathy (53.8%), bone fractures (42.3%), cancer-related fatigue (30.8%), and renal impairment (15.4%). In total, the patients received an average count of about two medical treatment modalities (SD =  ± 1). An overall assessment of disease severity in the SC could not be generated due to missing data in almost half of the patients. Table 1 lists all population characteristics arranged by cohort.

**Table 1:** Baseline characteristics of consecutive MM patients by cohort

Characteristic	SC (*N* = 26)	AC (*N* = 15)	EC (N = 8)
Gender, *N* (%)			
Male	16 (61.5)	10 (66.7)	6 (75.0)
Female	10 (38.5)	5 (33.3)	2 (25.0)
Distance to OTT, M (± SD) / Mdn (range)			
(km)	8.6 (± 5.9)	7.9 (± 5.9)	9.7 (2.2–26)
Anthropometrics, M (± SD) / Mdn (range)			
Age (years)	64 (± 8)	65 (± 8)	66.5 (62–79)
Weight (kg)	80 (± 18)	76 (± 15)	70 (59–103)
Height (cm)	173 (± 8)	170 (± 10)	171 (161–183)
BMI (kg/m^2^)	26 (± 5)	26 (± 4)	25 (20–26)
Paraprotein**,** *N* (%)			
IgA	5 (19.2)	4 (26.7)	2 (25.0)
IgG	13 (50.0)	8 (53.3)	4 (50.0)
Kappa light chains	1 (3.8)	0	0
No accessible information	7 (26.9)	3 (20.0)	2 (25.0)
Durie &Salmon stage [16], *N* (%)			
I	3 (11.5)	1 (6.7)	1 (12.5)
II	4 (15.4)	2 (13.3)	1 (12.5)
III	7 (26.9)	4 (26.7)	1 (12.5)
No accessible information	12 (46.2)	8 (53.3)	5 (62.5)
Total symptoms, M (± SD) / Mdn (range)			
	3 (± 1.6)	3.5 (1.7)	2.5 (1–5)
Symptoms, *N* (%)			
Bone lesions			
Focal	3 (11.5)	1 (6.7)	0
Disseminated	18 (69.2)	12 (80.0)	7 (87.5)
Bone fractures	11 (42.3)	7 (46.7)	4 (50.0)
Renal insufficiency	4 (15.4)	4 (26.7)	2 (25.0)
Fatigue	8 (30.8)	4 (26.7)	1 (12.5)
Polyneuropathy	14 (53.8)	8 (53.3)	3 (37.5)
Impairments			
Cognitive	6 (23.1)	4 (26.7)	1 (12.5)
Coordinative	6 (23.1)	3 (20.0)	1 (12.5)
Physical capacity	6 (23.1)	3 (20.0)	2 (25.0)
Immune function	4 (15.4)	2 (13.3)	0
Weight loss	6 (23.1)	4 (26.7)	2 (25.0)
No accessible information	1	0	0
Total comorbidities, M (± SD) / Mdn (range)			
	2 (± 1.6)	1.7 (± 1.7)	1 (0–3)
Comorbidities, *N* (%)			
Other cancers	2 (7.6)	1 (6.7)	1 (12.5)
Cardio-vascular	16 (61.5)	11 (73.3)	6 (75.0)
Respiratory	2 (7.7)	1 (6.7)	0
Metabolic	10 (38.5)	6 (40.0)	4 (50.0)
Orthopedic	4 (16.1)	0	0
Others	8 (30.7)	4 (26.7)	2 (25.0)
No accessible information	1	0	0
Total med.treatment modalities, M (± SD) / Mdn (range)			
	2.2 (± 1)	2.5 (± 0.8)	3 (2–4)
Received medical treatment, *N* (%)			
Chemotherapy	23 (88.5)	15 (100.0)	8 (100.0)
Surgery	11 (42.3)	8 (53.3)	5 (62.5)
Radiotherapy	13 (50.0)	10 (66.7)	7 (87.5)
Stem cell transplantation	9 (34.6)	5 (33.3)	2 (25.0)
No accessible information	1	0	0

*Abbreviations: AC*, adherence cohort; *BMI*, body mass index; *EC*, efficacy cohort; *Ig*, immune globulin; *M*, mean; *Mdn*, median; *N*, number of patients; *OTT*, oncologic exercise and movement therapy; *QS*, questionnaires; *SC*, safety cohort; *SD*, standard deviation

### Feasibility and exercise adherence

**Fig. 3:**
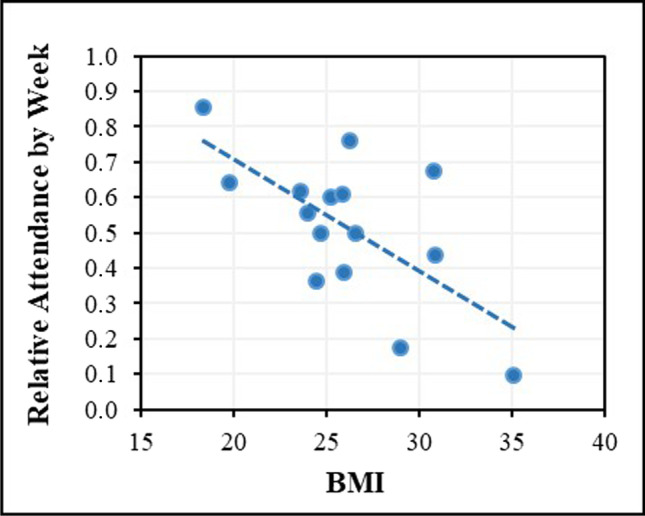
Pearson correlation of BMI (at baseline) and relative attendance by week of 15 consecutive patients (AC). Abbreviations: AC, adherence cohort; BMI, body mass index

Between January 2012 and September 2019, a total of 26 patients participated in exercise therapy at the OTT. Most patients commenced in 2018 (five patients), whereas in 2019 until September, only one patient registered at OTT. Six patients were still attending the OTT at the time of data collection. An overview of patient admission, exercise participation, and dropout are illustrated in Fig. 2. No adverse events were documented for any of the outlined patients. The adherence and attrition values are provided in Table 2. The feasibility analysis is conducted among the values for the SC. These reveal enormous heterogeneity in duration of participation in the OTT program as well as attendance by week and session. The average participation period amounts 75.5 weeks (SD = 81.1) at a tremendous range (3–251 weeks). Patients attend exercise between two and 151 weeks or between two and 269 sessions. On average, this results in 37 (SD =  ± 43.5) weeks or 58.1 (SD =  ± 75.2) exercise sessions. The corresponding relative exercise adherence in weeks (weeks with exercise divided by participation period) or sessions (exercise sessions divided by participation period) is 0.5 (SD =  ± 0.2) or 0.8 (SD =  ± 0.4), respectively. Half of the patients (*N* = 13) attend OTT for longer than 36 exercise sessions and about 70% (*N* = 18) for longer than 12 exercise sessions. Data on disease-related death of patients was not accessible. Although exercise adherence in terms of frequency and participation duration as well as early dropout was not a criterion for determining cohort allocation, the data in Table 2 clearly reveals that almost without exception all absolute and relative indicators of exercise adherence and attrition improve incrementally through the cohort reduction procedure of patient flow along with decreasing dispersion for the total observation period. Based on the normal distribution test (Shapiro–Wilk), correlation analyses were conducted according to Spearman or Pearson, respectively. At baseline, the factors BMI (*p* < 0.01), the number of comorbidities (*p* < 0.05), and moderate work-related activity (*p* < 0.05), as well as the scores for diarrhea (*p* < 0.05) and role functioning (*p* < 0.05) of the EORTC QLQ_C30, exhibited negative correlations with exercise adherence. Positive correlations were found for general fatigue (*p* < 0.05). All parameters show moderate to strong correlation according to Cohen [17], with BMI showing the strongest at *r*_p_ =  − 0.657 (*p* < 0.01) (Fig. 3). Noteworthy, only BMI and number of comorbidities correlate with a relative indicator of exercise adherence, thus in relation to the entire participation period. All the others exclusively show correlations with absolute criteria for exercise adherence measured in weeks or sessions. In addition, group comparisons were calculated based on the dichotomous variables bone fractures and surgery, which were approximately equally distributed among participants (Table 1). Both grouping variables yielded no statistically significant results.

**Table 2:** Adherence and attrition by cohort and observation period

	SC	AC	EC	
	Total (*N* = 26)	Total (*N* = 15)	Total (*N* = 8)	MP1**–**MP2 (*N* = 8)
Participation**/**observation period, M (± SD) / Mdn				
By week				
	75.5 (± 81.1)	98.5 (± 84.6)	91.5	20
* Range*	*3–251*	*8–251*	*28–251*	*8–46*
By session				
	*–*	*–*	*–*	20
* Range*	*–*	*–*	*–*	*16–30*
Attendance, M (± SD) / Mdn				
By week				
Absolute	37.0 (± 43.5)	51.8 (± 50.1)	64	13.5
* Range*	*2–151*	*3–151*	*17–151*	*8–28*
Relative^1^	0.5 (± 0.2)	0.5 (± 0.2)	0.6	0.6
* Range*	*0.1–0.9*	*0.1–0.9*	*0.4–0.8*	*0.48–1*
By session				
Absolute	58.1 (± 75.2)	84.3 (± 88.5)	116	20
* Range*	*2–269*	*4–269*	*27–269*	*16–30*
Relative^2^	0.8 (± 0.4)	0.8 (± 0.4)	1	1
* Range*	*0.1–1.5*	*0.1–1.5*	*0.4–1.5*	*0.5–2.1*
Still attending, *N* (%)				
	6 (23.08)	6 (40.0)	5 (62.5)	5 (62.5)
Dropout, *N* (%)				
By attended weeks				
< 6 Weeks	4 (15.4)	1 (6.67)	0	–
< 12 Weeks	8 (30.8)	2 (13.3)	0	–
By attended sessions				
< 12 Sessions	8 (30.8)	2 (13.3)	0	–
< 36 Sessions	13 (50.0)	6 (40.0)	2 (25.0)	–

^1^Divided by participation period

^2^Divided by attended weeks

*Abbreviations: AC*, adherence cohort; *EC*, efficacy cohort; *SC*, safety cohort; *M*, mean; *Mdn*, median; *MP*, measurement point; *N*, number of patients; *SD*, standard deviation

### Exercise efficacy

**Fig. 4:**
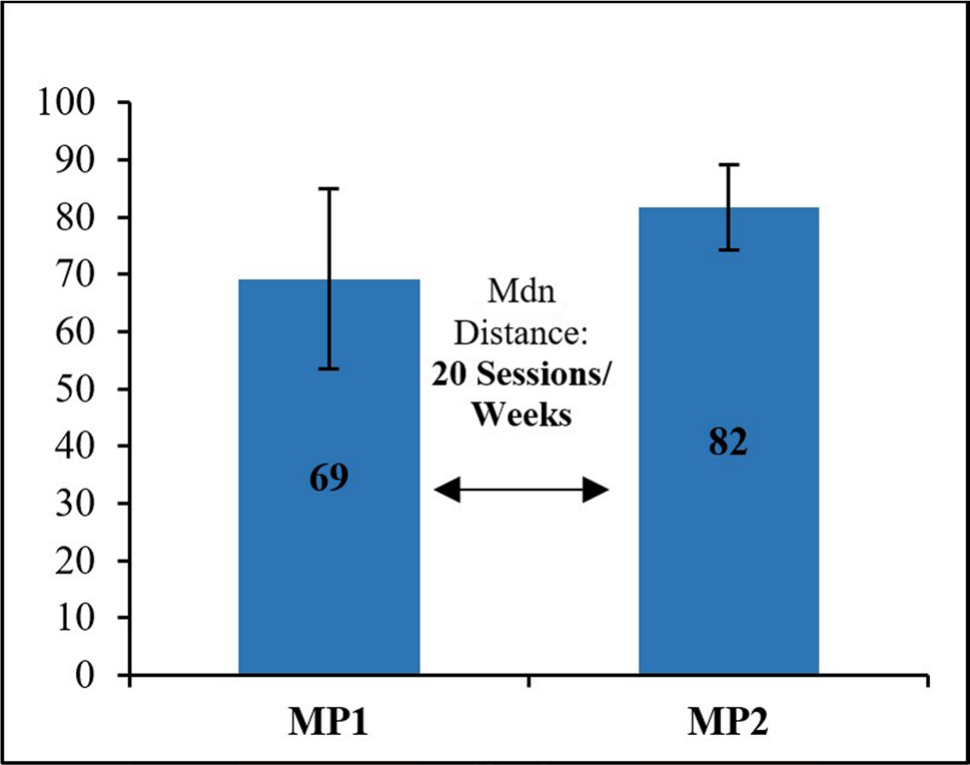
Mean values of physical functioning scores (EORTC QLQ_C30) for MP1 and MP2 of 8 consecutive patients (EC). Scores of the EORTC QLQ_30 range from 0 to 100. Sessions between MPs: Mdn = 20; range = 16–30; weeks between MPs: Mdn = 20; range = 8–46. Abbreviations: EC, efficacy cohort; Mdn, median; MP, measurement point

The time comparison for two MPs was conducted among patients of the EC via *t*-test or Wilcoxon depending on results of the distribution test (Shapiro–Wilk). The median distance between the MPs was 20 exercise sessions (range: 16–30) or 20 weeks (range: 8–46), respectively (see Table 2). Significant or nearly significant results were exclusively found for scales of the EORTC QLQ_C30. The *t*-tests yielded a significant result for the physical functioning scale score, *t*(7) = 2.934, *p* = 0.022, indicating an improvement (see Fig. 4). Moreover, a trend towards significant improvement could be observed via results of the Wilcoxon test for the fatigue scale score, *z* =  − 1.76, *p* = 0.078.

## Discussion

### Feasibility

Twenty-six patients commenced exercising at the OTT between January 2012 and November 2019 without active acquisition. No adverse events occurred. Mortality data were not accessible. However, considering the low 5-year relative survival rate of MM patients [18] (www.krebsdaten.de; [18]: Zentrum für Krebsregisterdaten > Krebsarten > Multiples Myelom), the dropout rates in the study population which range between 25.4% (dropout within 6 weeks of exercise), 30.2% (dropout within 12 weeks of exercise/less than 12 sessions), and 50% (less than 36 sessions, might have been affected by mortality). Additionally, previous studies systematically excluded patients at increased risk of bone fractures [10]. In the present study, at baseline, a high percentage of patients exhibited disseminated bone lesions (69.2%) and bone fractures (42.3%, not at risk for instability) (Table 1). The impact of these factors on admission rate and exercise adherence remains speculative due to the real-life nature of the data and the missing data of determinants for early dropout. However and most importantly, the real-life data in the present research confirm safety and feasibility of exercise therapy with MM patients in clinical settings.

### Adherence and efficacy

Absolute indicators of exercise adherence such as participation period and total attendance by week and session are indicators for sustained participation or durability. Relative values indicate exercise frequency. Most strikingly the level of BMI is negatively associated (*p* < 0.01) with exercise frequency by week. A high BMI might indicate psycho-social as well as physical barriers to regular physical activity, such as a generally rather unfavorable health-related lifestyle, lower motivation, and affinity for exercising as well as increasing risks for serious systemic secondary diseases [19]. In contrast, absolute indicators of exercise adherence are not significantly correlated with BMI. Additionally, Fig. 3 illustrates extreme outliers with three BMI values above 30 and two under 20 accounting for one-third of the sample size while two-thirds of the values scatter around a BMI of 25, suggesting a statistical distortion effect. A high diarrhea (single-item) and a high role functioning scale score (multi-item; e.g., “Have you been restricted at your work or at other daily activities?”) reflect the disability of MM patients and might pose additional obstacles to a long-term maintenance of an exercise routine as indicated by the negative correlations with absolute adherence values. Significant negative correlations found for moderate work-related activity and absolute indicators of exercise adherence are most likely caused by an overrepresentation of zero values as only six out of 15 patients exhibit available data on that item. Moreover, the limited informative value of the GPAQ, particularly in patients with cancer, has already been reported [20]. The positive correlation of fatigue and participation period, with respect to existing research, might depict an interesting and promising finding. Cancer-related fatigue is often perceived as one of the highest burden of MM patients [21, 22] and can be mitigated by exercise more effectively than by drug therapy [23]. A recently published meta-analysis confirms this effect in hemato-oncological entities [24]. Accordingly, high fatigue burden and efficacy of reduction through exercise might positively impact long-term exercise motivation with a positive trend seen in the EC (*N* = 8).

The associations reported in the literature between bone fractures, major surgery, and low training adherence [21, 25] could not be supported due to non-significant results via grouping tests for surgery and bone fractures. Furthermore, the movement-reducing effect of stem cell transplantation [26, 27] could not be investigated due to the small sample size and the unequal distributed grouping variable (see Table 1).

The physical tests yielded hardly evaluable data also because of the heterogeneity of the performed strength tests among patients and MPs. Consequently, comparability was hardly producible. The application of a feasible and quick test like the handgrip strength test could solve this problem in future studies.

The most interesting and promising result of this investigation is the statistically significant change in physical functioning over a training period of 16 to 30 exercise sessions between MP1 and MP2 in the EC (*N* = 8). Existing interventional studies point in a similar direction regarding beneficial effects on physical functioning by exercising in MM patients [9, 14, 25, 27]. Nevertheless, there are several remarkable factors with respect to this result. First, the present exercise period between patients of the EC is very heterogeneous and significantly shorter than in existing interventional studies on exercise therapy in MM patients [9]. Furthermore, the result is based on questionnaire data only and has not been objectively measured via physical assessment such as 30 s sit-to-stand-test (30STS). Most importantly, accounting for the small sample size of eight patients and the heterogeneous exercise participation and measurement procedure, either a large effect size or factors of chance and positive selection bias led to statistical significance. Recently, we demonstrated a highly significant improvement of physical activity and functioning in patients with the precursor condition monoclonal gammopathy of undetermined significance under whole-body vibration exercise training [28].

Notwithstanding the promising nature of some findings, there are important limitations particularly resulting from the retrospectivity of the conducted evaluation procedure comprising the applied patient flow allocation to different cohorts and the post hoc definition of applied statistical measures, as well as generally from the real-life nature of the data evaluated. The allocation of patient flow to specific cohorts was carried out to enable cross-sectional and longitudinal data evaluation. Although the number of realized exercise sessions between measurements was not a relevant allocation criterion, the applied procedure resulted in an unintended incremental increase of mean exercise adherence by absolute and relative indicators among cohorts (Table 2), and therefore, most likely led to a positive selection bias. Additionally, the consecutive reduction in cohort sizes limits the statistical representation of the underlying total population of MM patients restraining the range of findings. Moreover, defining statistical measures post hoc generally leads to an incalculably size of alpha error, hence, increasing likelihood for coincidental findings. In conclusion, the retrospective of the present evaluation can only provide indications of possible associations regardless of the applied statistical measures. Additional limitations result from the real-life nature of the present study. Firstly, it cannot be precluded that a distortion of the patient selection already existed at the time of admission to the OTT. It remains unclear whether all eligible patients were informed and basically had access to the OTT or whether the participating patients already possessed certain attributes that did not adequately reflect the characteristics of the underlying population (e.g., motivation and training affinity). Furthermore, there is no precise knowledge of possible confounders during exercise therapy such as disease or psycho-social burdens. Recapitulatory, the real-life findings resulting from the reduced cohorts on adherence and efficacy are clinically relevant and offer some important indications for future research. In order to improve the quality of future research without reducing clinical relevance, the existing measurement procedures should be supplemented by additional subjective measurements (mainly questionnaires) filled out at home, and on-site training could be expanded with supervised online training. Possibly, this will reduce the heterogeneity of measurements and exercise adherence by reducing the barriers for participation. Furthermore, we highly recommend the implementation of a routinely evaluation design under previously defined statistical measures to enhance the scientific range of real-life data investigations.

## Conclusion

The present observatory study with MM patients reveals safety and feasibility while indicating adherence and efficacy under real-life therapy circumstances in an oncologic care structure. Reasons for the high dropout rate as well as the low average exercise frequency cannot be determined. Although clinically highly relevant, found obstacles to exercise adherence, as well as improvements in questionnaire scale scores, need to be further investigated in confirmatory study designs.

## Data Availability

The data sets generated during and/or analyzed during the current study are available from the corresponding author on reasonable request.
